# Comparative Membrane-Associated Proteomics of Three Different Immune Reactions in Potato

**DOI:** 10.3390/ijms19020538

**Published:** 2018-02-10

**Authors:** Dharani Dhar Burra, Marit Lenman, Fredrik Levander, Svante Resjö, Erik Andreasson

**Affiliations:** 1 Department of Plant Protection Biology, Swedish University of Agricultural Sciences, 230 53 Alnarp, Sweden; Dharani.Burra@gmail.com (D.D.B.); marit.lenman@slu.se (M.L.); svante.resjo@slu.se (S.R.); 2 Department of Immunotechnology, Lund University, 221 00 Lund, Sweden; fredrik.levander@immun.lth.se

**Keywords:** ETI, effector-triggered immunity, PTI, potato, proteomics, Désirée

## Abstract

Plants have evolved different types of immune reactions but large-scale proteomics about these processes are lacking, especially in the case of agriculturally important crop pathosystems. We have established a system for investigating PAMP-triggered immunity (PTI) and two different effector-triggered immunity (ETI; triggered by Avr2 or IpiO) responses in potato. The ETI responses are triggered by molecules from the agriculturally important *Phytophthora infestans* interaction. To perform large-scale membrane protein-based comparison of these responses, we established a method to extract proteins from subcellular compartments in leaves. In the membrane fractions that were subjected to quantitative proteomics analysis, we found that most proteins regulated during PTI were also regulated in the same way in ETI. Proteins related to photosynthesis had lower abundance, while proteins related to oxidative and biotic stress, as well as those related to general antimicrobial defense and cell wall degradation, were found to be higher in abundance. On the other hand, we identified a few proteins—for instance, an ABC transporter-like protein—that were only found in the PTI reaction. Furthermore, we also identified proteins that were regulated only in ETI interactions. These included proteins related to GTP binding and heterotrimeric G-protein signaling, as well as those related to phospholipase signaling.

## 1. Introduction

Plants possess an intriguing and unique immune system that is different from many other living organisms. Despite these differences, the innate immune system of plants performs similar functions as that in animals [1]. Plant immune responses can be broadly categorized into PAMP-triggered immunity (PTI) and effector-triggered immunity (ETI) [2]. PAMPs (pathogen-associated molecular patterns) are surface exposed, pathogen-associated molecules that are generally conserved across microbial kingdoms. Plants detect PAMPs via membrane-bound PRRs (pattern recognition receptors) that often have a kinase domain. PAMP recognition leads to molecular responses such as reactive oxygen species (ROS) production, MAP kinase and transcription factor activation, followed by defense gene expression [3]. Through evolution, however, successful pathogens have evolved to produce effector molecules that interfere with PTI responses, enabling successful infection. This is known as effector-triggered susceptibility (ETS) [2]. In response to this, plants have evolved the ability to counter effector molecules via the production of specialized resistance proteins (R-proteins). R-proteins recognize the presence of specific effectors (mainly in the cytoplasm). This interaction and the associated molecular reactions constitute ETI [2]. Phenotypically, ETI defense responses are typically associated with a specialized form of programmed cell death (PCD) known as the hypersensitive response (HR) [2,4], resulting in the arrest of pathogen spread and infection.

Recent evidence from a wide variety of plant pathogen systems has indicated that PTI and ETI are mutually not exclusive. PAMPs can trigger typical ETI responses, while the effects of effectors are not restricted to ETI responses [5]. For example, INF1, a PAMP in *Phytophthora infestans*, causes cell death when expressed in *Nicotiana benthamiana*, a phenotype associated with an ETI response [6]. However, like other PAMPs, INF1 is also recognized by a PRR in potato [7], indicating a continuum between PTI and ETI. Large-scale transcriptomic investigations in Arabidopsis have also indicated that there is an overlap in ETI and PTI signaling [8]. Recently, Pombo et al. [9] used the tomato–*Pseudomonas syringae pv. tomato* pathosystem to identify genes induced specifically in bacterial ETI and PTI responses. In this study, the authors were able to identify an overlap between these two processes. Using this approach, they were also able to identify an ETI-specific tomato protein kinase *Epk1*, which when silenced transiently in *Nicotiana benthamiana* resulted in delayed PCD and compromised resistance to *Pseudomonas syringae pv. tabaci*.

Genome analysis of the devastating pathogen *Phytophthora infestans* shows an expansion in effector coding genes [10]. Two well-known *P. infestans* effectors are IpiO and Avr2, both of which interact with characterized resistance genes. In potato plants carrying the *Solanum bulbocastanum* resistance gene *Blb1*, IpiO acts as an avirulence protein, an interaction that leads to an HR formation [11]. Likewise, Avr2 also elicits HR in plants containing a resistance protein belonging to the *R2* NB-LRR gene family [12,13,14]. In addition, Avr2 has been shown to associate with BSU-like protein 1 (BSL1) [15], which is thought to be involved in brassinosteroid-associated signal transduction [16]. Additionally, Saunders et al. [15] showed that perception of Avr2 by R2 is dependent on BSL1.

Most of the previous studies have been carried out at the transcriptomic level. However, the correlation between mRNA and protein abundance is limited and this could be due to, for example, differences in protein translational efficiency, protein stability, or protein transport. In the mammalian system, the correlation factor (R^2^) is only 0.41 [17], and evidence in potato under pathogen stress suggests that gene expression only correlates with protein abundance in approximately half of the induced transcripts and peptides [18]. Correlations between protein levels and mRNA transcript abundance vary across conditions, with higher coherence in levels observed in steady state as opposed to during a stress response [19]. Hence, a large-scale proteomics-based approach can, therefore, expand our understanding of stress responses, such as those linked to plant defense and immunity. However, large-scale protein level studies with regard to exclusivity and commonality between PTI- and ETI-associated molecular signaling in an agriculturally important crop–pathogen system, such as in the potato–*Phytophthora infestans* interaction, does not exist. Furthermore, no comparative study on protein level information on ETI responses caused by different effector/R-protein combinations is available, and generally very little is known about membrane-enriched fractions in this biological context.

In this study, we performed a quantitative proteomics study of PTI and ETI responses in potato using a membrane-enriched fraction after establishing this fractionation method in intact plants. As a model for PTI responses, we used potato leaves infiltrated with *Agrobacterium*-containing empty vector. As models for ETI, we investigated responses after *Agrobacterium*-mediated expression of two *P. infestans* effectors (IpiO and Avr2) in potato plants expressing the corresponding R-proteins. *Agrobacterium* is known to contain many different PAMPs and has been used as an inducer of PTI with reduced subsequent pathogen infection [20,21,22,23]. Disarmed *Agrobacterium* infiltration is further known to activate transcription of pathogenesis-related genes and accumulation of pathogenesis-related proteins [24,25]. By using this setup, we were able to do a comparative analysis of three different immune responses. 

## 2. Results and Discussion

### 2.1. Phenotypes of PAMP-Triggered Immunity (PTI) and Effector-Triggered Immunity (ETI) Responses

Disarmed *Agrobacterium*-infiltrated wildtype cv. Désirée potato plants were used to study PTI responses and were compared with samples from two different ETI responses. The first ETI model was Blb1-containing Désirée infiltrated with *Agrobacterium* transformed with the IpiO effector gene, and the second was R2-containing Désirée infiltrated with *Agrobacterium* transformed with Avr2 effector gene. The infiltrated samples were subjected to phenotypic analysis at 18 hpi (hours post infiltration) and 3 dpi (days post infiltration). 

**Figure 1:**
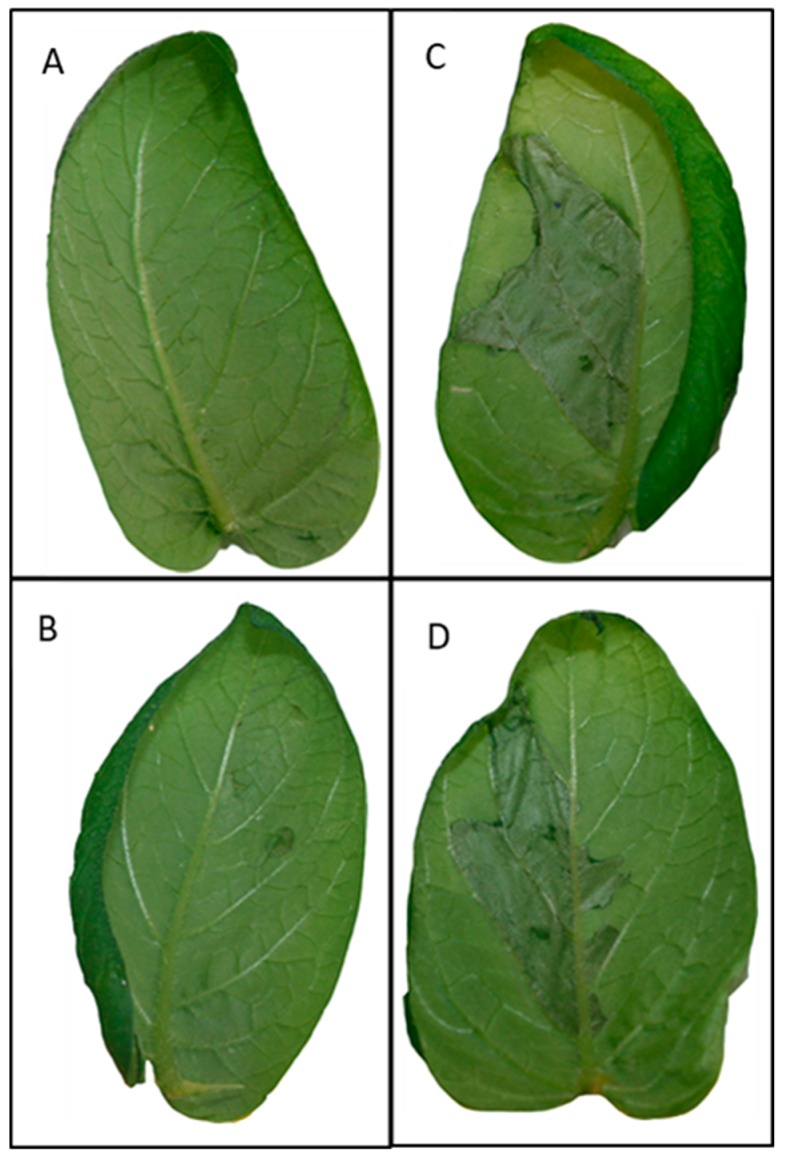
Potato leaflets 3 days post inoculation. (**A**) Control leaves: Désirée leaflets infiltrated with only infiltration media; (**B**) PAMP-triggered immunity (PTI) model: Désirée leaflets infiltrated with *Agrobacterium* carrying an empty vector; (**C**) Effector-triggered immunity (ETI)-Avr2 model: stable transgenic Désirée carrying the *R2* resistance gene infiltrated with *Agrobacterium* carrying the *Avr2* effector gene; (**D**) ETI-IpiO model: stable transgenic Désirée carrying the *Blb1* resistance infiltrated with *Agrobacterium* carrying the *IpiO* effector gene.

None of the infiltrated samples showed visible phenotypic symptoms at 18 hpi. Three days post infiltration, a strong reaction and even cell death coinciding with the infiltration area were found in both the ETI interactions (Figure 1C,D). Both these interactions had similar degrees of cell death at the whole infiltrated area. In the PTI interaction, small areas of cell death were observed occasionally in the zone of infiltration (Figure 1B). No response was identified in cv. Désirée leaflets infiltrated with the infiltration medium only (Figure 1A).

### 2.2. Subcellular Protein Fractionation

**Figure 2:**
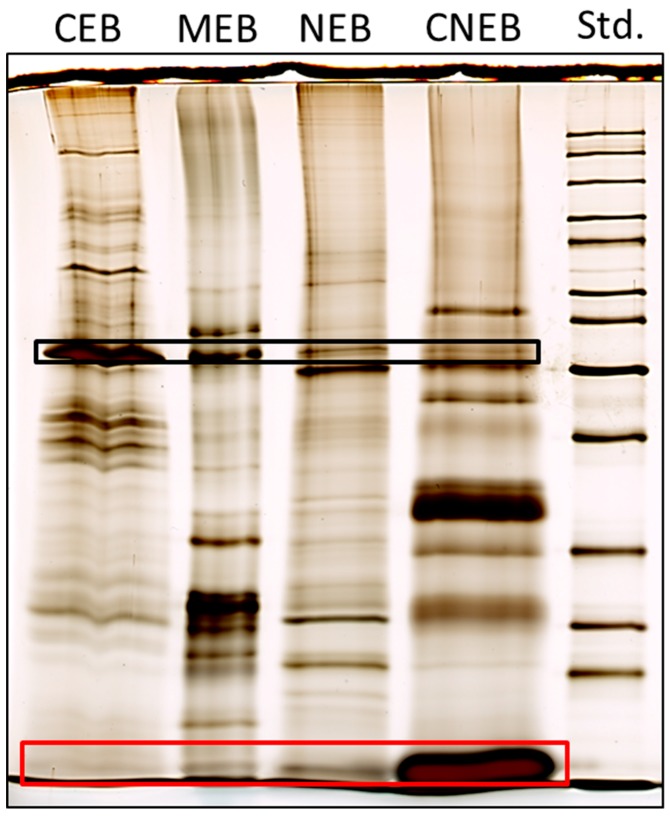
SDS-PAGE analysis of various subcellular fractions. CEB corresponds to proteins from the cytoplasmic fraction; MEB corresponds to proteins from the membrane-associated fraction; NEB corresponds to proteins from the nuclear-associated fraction; and CNEB corresponds to proteins associated with chromatin. Std.(standard) corresponds to the size marker. Bands marked within the black lined box correspond to the size of the large subunit of rubisco. The band marked within the red lined box corresponds to the size of histones.

**Figure 3:**
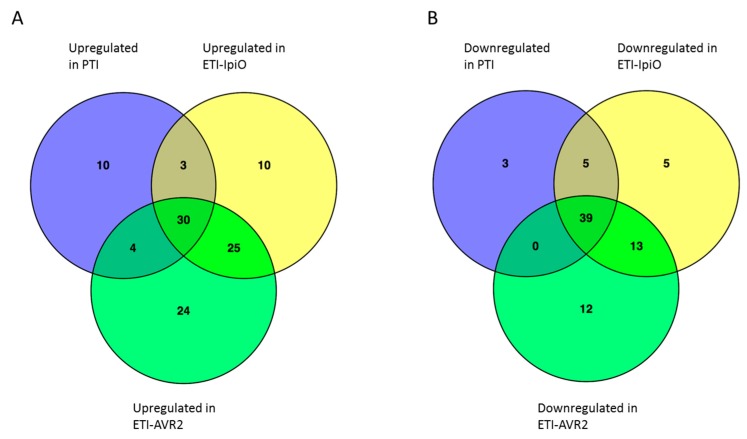
Analysis of the fraction of membrane-associated proteins. Number of proteins significantly regulated in PTI and the two ETI conditions (Blb1-IpiO and AVR2-R2). (**A**) shows upregulated proteins, and (**B**) shows downregulated proteins.

The fractionation procedure was based on successive centrifugation steps, wherein the supernatant at each step was extracted in a different buffer leading to four different buffers containing four protein fractions named as follows: cytoplasmic (CEB), membrane (MEB), soluble-nuclear (NEB), and chromatin-bound (CNEB). In order to identify whether differences existed in the banding pattern between the four fractions, each fraction was analyzed on an SDS-PAGE gel (Figure 2). The banding patterns of the four fractions were clearly different, indicating that the subcellular fractionation procedure had resulted in the isolation of different protein fractions. The CNEB fraction contained a very prominent band corresponding to the large Rubisco subunit (Figure 2). Rubisco is one of the most abundant proteins in plant tissues and is associated with the stromal component of chloroplasts [26]. The CNEB fraction contained a strong band corresponding to histones (Figure 2); this indicates that proteins associated with chromatin are indeed extracted in the CNEB fraction. The total amount of protein obtained from the different fractions also differed. The different lanes on the gel contain approximately equal amounts of protein in order to better display the differences in banding patterns. On average, a total of 470 µg was obtained from the CEB fractions, 150 µg from the MEB fractions, and 5 µg from the CNEB fractions. Since the MEB fraction seemed to contain large amounts of potentially interesting proteins, and little is specifically known about this fraction from plants in relation to immunity, this fraction was chosen for further analysis. The protein abundances in our 18 h PTI model and the two ETI models were compared using potato leaves infiltrated with only infiltration medium as control (Figure 3, Supplementary Tables S1 and S2). 

### 2.3. Membrane-Associated Proteins in the PTI Response

In the quantitative analysis of the PTI interaction, 585 proteins were used. The fraction contained predominantly chloroplastic, ribosomal, and mitochondrial proteins. In the PTI condition, 47 proteins were downregulated and 47 proteins were upregulated (Figure 3, Table 1). Among the downregulated proteins (Supplementary Tables S1 and S2), a large number were chloroplast proteins involved in photosynthetic processes, such as chlorophyll a/b binding proteins, photosystem proteins, NAD(P)H-quinone oxidoreductases, and cytochrome bf-6 complex components. In total, 26 of the downregulated proteins are involved in photosynthesis. This is consistent with the well-established observation that infection results in the downregulation of components of the photosynthetic machinery [27].

Among the other downregulated proteins was a plasma membrane-associated temperature-induced lipocalin [28]. In Arabidopsis, temperature-induced lipocalins have been implicated in moderating tolerance to oxidative stress [29]. Another protein, a bacterioferritin homolog, was also downregulated. The closest Arabidopsis homolog is a peroxiredoxin Q. Similar to lipocalins, peroxiredoxins are also involved in protection against oxidative stress [30]. These results indicate that some components related to oxidative stress tolerance are downregulated during PTI.

The upregulated proteins during PTI were more varied in function than the downregulated proteins and are listed in Table 1. A number of ATP synthases were upregulated. This might reflect an increased need for energy for the activation of defenses [31]. Interestingly, an LRR-like receptor protein kinase (LRR-RK) was found to be upregulated. Expression of the orthologous Arabidopsis transcript has been hypothesized to correlate with auxin levels in Arabidopsis [32]. Another protein annotated as translationally-controlled tumor protein homolog was also upregulated. This protein has been shown to be upregulated in Arabidopsis in response to effectors produced by the Gram-negative bacterium *Pseudomonas syringae pv. tomato* [33]. The TAO1 (target of AvrB operation) protein that is necessary for *Pseudomonas syringae* AvrB-triggered resistance [34] was also upregulated.

Other proteins upregulated during PTI were glycolate oxidase and a peroxidase. Glycolate oxidase has been shown to generate hydrogen peroxide during stress [35]. Plant peroxidases belong to the PR9 family of PR proteins. They use hydrogen peroxide to catalyze the oxidation of a number of different substances [36]. The protein MAR binding filament protein (MFP1), which has been previously shown to be induced in tomato in response to the elicitor COS-OGA [37], was also upregulated. Treatment of Arabidopsis suspension cells and protoplasts with COS-OGA also generates hydrogen peroxide [38]. An ABC transporter-like protein that has been shown to be induced in response to oxidative stress [39] was also upregulated specifically in PTI. Therefore, the abovementioned proteins seem to be involved in reactive oxygen species (ROS) signaling.

### 2.4. Proteins in ETI Responses

Seventy-four proteins were downregulated and 92 proteins were upregulated from one or both of the ETI interactions (Figure 3). Proteins upregulated in the ETI interactions are mentioned in Table 2. There was a substantial overlap with the proteins regulated in the PTI condition, particularly among the downregulated proteins (Figure 3). Thus, out of the proteins downregulated in PTI, only 3 were uniquely downregulated, and all of the downregulated proteins discussed in the PTI section above were also downregulated in the ETI conditions. In addition, 30 more proteins were downregulated in ETI (Figure 3). Ten of these were chloroplast proteins involved in photosynthesis, as discussed in the PTI section. 

The upregulated proteins in ETI overlapped with those upregulated in PTI, but less so than the downregulated proteins. Of the proteins upregulated in PTI, 10 were uniquely upregulated in that condition. Thirty-seven proteins were upregulated in both PTI and ETI and 59 were significantly upregulated in only ETI (Figure 3; Table 2); these latter included several proteins that showed the same tendency in all three sample types but did not reach the significance level in PTI. Among the proteins that were regulated significantly in both ETIs, a number were ribosomal proteins; possibly reflecting increased overall protein synthesis during this phase. Two superoxide dismutases were also upregulated. Superoxide dismutase catalyzes the dismutation of the superoxide radical. Their role is probably to protect the plant against the reactive oxygen species produced during the oxidative burst [40]. A further indication of active protective mechanisms to ROS is indicated by the upregulation of a chloroplastic lipocalin in ETI-IpiO, which has previously been shown to be involved in modulating tolerance to oxidative stress [29]. Interestingly, a lipocalin was downregulated in PTI (see above). A GTP-binding protein Era and an ethylene-responsive small GTP-binding protein were upregulated in the ETI interactions. A prominent role of regulation of plant immunity by GTP binding proteins has been suggested [41] and, based on our observations, it is tempting to speculate that GTP proteins might be specifically related to ETI plant immunity.

Phospholipase A1 was upregulated only in ETI-Avr2. This protein belongs to a class of DAD (defective in anther dehiscence)-like proteins that is involved in jasmonic acid (JA) synthesis [42], possibly indicating a role for JA-mediated molecular signaling in Avr2-induced ETI. A heat shock protein 70-3 was also specifically upregulated in ETI-Avr2. This protein might connect oxidative stress induction and G-protein-dependent signaling in this ETI interaction as it has been shown to be involved in cGMP-dependent stress responses to hydrogen peroxide production [43] and again might underpin the involvement of GTP/GMP signaling in ETI. In comparison, a serine/threonine protein kinase was specifically upregulated in ETI-IpiO. The closest Arabidopsis homolog of this protein is annotated as an STN7 protein kinase, and it has been shown to link photosynthetic activity to ROS-induced molecular signaling during stress [44]. In combination with our observations with regards to chloroplastic lipocalin, upregulation of STN7 further supports the observation that ROS protection mechanisms might be necessary for ETI, specifically.

## 3. Materials and Methods

### 3.1. Plants and Agrobacterium Inoculation

Three sets of *Solanum tuberosum* (cv. Désirée) wildtype plants, AO1-22 (Désirée carrying Rpi-Blb1 resistance gene) [45,46] and T16 (Désirée plants carrying R2-type resistance gene) [13] were grown according to Abreha et al. [45]. Plants were initially grown in vitro on Murashige–Skoog (MS) media with vitamins in controlled growth conditions with 16 h light, day temperature of 23 °C and night temperature of 18 °C for 2 weeks. The plantlets were then transferred to soil and grown for 4 more weeks at approximately 22 °C with a cycle of 16 h of light and 8 h of darkness. The plants were supplemented with fertilizer (Rika S, SW Horto, Hammenhög, Sweden) once every second week. *Agrobacterium* strain AGL1 transformed with either an empty vector, IpiO effector gene, or Avr2 effector gene were grown according to Du et al. [47]. All antibiotics were used at a final concentration of 25 μg/mL except of spectinomycin that was used at a final concentration of 100 μg/mL. *Agrobacterium* strains were grown in 10 mL YEB medium supplemented with 1 μL acetosyringone (200 mM), 100 μL of 1 M MES buffer and appropriate antibiotics. The cultures were grown for 24 h at 28 °C, 200 rpm until OD_600_ reached 1. The bacteria were harvested from the YEB medium by centrifuging at 3000× *g* for 10 min. The bacterial pellet was re-suspended in infiltration medium MMA medium (5 g/L MS salts, 1.95 g/L MES, 20 g/L sucrose, 200 μM acetosyringone, pH 5.6) to an OD_600_ of 0.3. For infiltrations, the abaxial surface of a minimum of 5 leaflets on each plant was infiltrated using a 5 mL needleless syringe. A total of 4 plants belonging to each genotype (wildtype, AO1-22, and T16) were infiltrated. Three days post infiltration (dpi), a minimum of 1 leaflet from each plant (total 4 plants) was used to assess macroscopic cell death phenotype. The complete experiment was repeated twice.

### 3.2. Subcellular Protein Fractionation

Out of the four infiltrated plants belonging to each genotype, two plants were sampled for protein extraction at 18 hpi (hours post infiltration). Two leaflets from each plant were sampled for subcellular protein fractionation. From each infiltrated leaflet, two samples were taken, each containing two stabs (corresponding to 100 mg fresh weight) from the infiltrated area. In summary, four samples (containing two stabs each) were obtained from each genotype. Each sample was put in a 1.5 mL Eppendorf tube with sea sand on ice before further sample processing. The whole experiments were carried out twice and resulted in eight samples of each type. Subcellular protein fractionation into cytoplasmic (CEB), membrane (MEB), soluble-nuclear (NEB), and chromatin-bound (CNEB) fractions was performed using a Subcellular Protein Fractionation Kit for Tissues (ThermoFisher Scientific; Waltham, MA, USA, Catalog No. 87790) with minor modifications (see below). Phosphatase inhibitors (5 mM sodium phosphate, 50 μM sodium orthovanadate, and 10 nM calyculin A) were added to each buffer before use. Briefly, proteins were extracted in four different buffers consecutively and final supernatants were frozen at −80 °C until further use. 

Each leaf sample was disrupted using pestle sticks in 1 mL ice-cold CEB. The sample was then passed through a tissue and centrifuged at 500× *g* for 5 min at 4 °C. The supernatant was cleared by re-centrifugation at 16,000× *g* for 10 min at 4 °C and saved as the cytoplasmic fraction. The 500× *g* CEB pellet was washed and centrifuged once with CEB and ice-cold MEB was added to the washed pellet. The pellet was then vortexed and incubated at 4 °C for 10 min with gentle mixing. After incubation, the solution was centrifuged at 3000× *g* for 5 min. The supernatant was cleared by re-centrifugation at 16,000× *g* for 10 min at 4 °C and the supernatant saved as the membrane fraction. The pellet obtained after the 3000× *g* centrifugation was washed once with MEB and centrifuged. To the resulting pellet, ice cold NEB was added, the sample was vortexed and incubated for 30 min at 4 °C with gentle mixing. After incubation, the sample was centrifuged at 5000× *g* for 5 min at 4 °C and the supernatant saved as the nuclear extract. The pellet obtained after the 5000× *g* centrifugation was washed and centrifuged once with NEB, and to the pellet room-temperature CNEB was added. The pellet was vortexed at maximum for 15 s and the sample was incubated at 37 °C for 15 min. After the room temperature incubation, the sample was centrifuged at 16,000× *g* for 5 min. The supernatant was defined as the chromatin sample.

### 3.3. Protein Concentration Determination

Protein concentration determination was performed using the bicinchoninic acid assay (Pierce™ BCA Protein Assay Kit, Thermo Fisher Scientific, Waltham, MA, USA; Catalog number: 23225) according the manufacturer’s instructions. The buffer for each fraction (CEB, MEB and CNEB) was used to dilute the standard curves for each type of the three sample types. 

### 3.4. Silver Staining

SDS-PAGE gels were stained with silver according to Blum et al. [48]. Briefly, gels were fixed in 40% ethanol, 10% acetic acid overnight. The gels were washed 3 times in water (20 min per wash). They were then incubated in 0.02% Na_2_S_2_O_3_ for 1 min, washed 3 times in water (1 min per wash), and incubated for 20 min in 0.2% AgNO_3_, 0.02% formaldehyde. After this incubation, the gels were washed twice in water (1 min per wash) and developed in a solution of 3% Na_2_CO_3_, 0.05% formaldehyde, and 0.0005% Na_2_S_2_O_3_. The development process was stopped by washing once with water (1 min) and then incubated in 0.5% glycine solution.

### 3.5. Tryptic Digestion and Mass Spectrometry

Proteins from the analyzed fractions were separated on a 14% SDS-PAGE gel. The entire lane was excised, washed, and the proteins digested with trypsin (Promega Trypsin Gold, Madison, WI, USA, Mass Spectrometry Grade Trypsin Gold, Catalog number: V5280). The tryptic digests were desalted using C18-based spin columns (The Nest Group, Inc., Southborough, MA, USA) as described in Chawade et al. [49]. Tryptic digests were subjected to HPLC-MS/MS analysis using an Eksigent nanoLC2D HPLC system connected online-with an LTQ Orbitrap XL ETD. The peptide samples were loaded onto an Agilent Zorbax 300SB C18 (0.3 mm ID, 5 mm, 5 µm particle size) pre-column and separated on an in-house packed PicoFrit column (Santa Clara, CA, USA; Agilent Zorbax 300SB C18, 75 µm ID, 150 mm, 3.5 µm particle size). The analytical column was pre-equilibrated with a buffer consisting of 0.1% formic acid (FA) in 5% ACN for 10 min at a flow rate of 10 µL/min, and peptide separation was conducted in 0.1% FA buffer using a 55 min linear gradient from 5% to 40% ACN, followed by a 5 min linear gradient from 40% to 80% ACN, at a flow rate of 350 nL/min. The eluted peptides were analyzed using an LTQ Orbitrap XL ETD. The Orbitrap was operated in data-dependent mode with survey scan spectra 400–2000 Da in the Orbitrap mass analyzer at target resolution 60,000, followed by selection of the seven most intense ions for fragmentation in the LTQ, using a mass window of 2 Da for precursor ion selection. The precursor ions were fragmented with normalized collision energy of 35 (with activation Q set to 0.25 and an activation time of 30 ms). Dynamic exclusion with a repeat count of 2 and a repeat duration of 20 s and exclusion duration of 120 s were used, with an exclusion list size of 499 and a 10 ppm relative exclusion mass width. 

### 3.6. Data Analysis

The raw data from the Orbitrap was converted to Mascot generic files (mgf) using ProteoWizard [50]. The Proteios software environment [51] was used to search the mgf files in Mascot version 2.3.01. The mgf files were searched against a database consisting of *Solanum* proteins from UniProt (www.uniprot.org), downloaded 24 August 2011; protein sequences from the Potato Genome Project [52] and the *Agrobacterium* proteins from UniProt, downloaded 10 March 2015, concatenated with an equal size decoy database (random protein sequences with conserved protein length and amino acid distribution, in total 36,512 target and decoy protein entries) generated using a modified version of the decoy.pl script from MatrixScience (http://www.matrixscience.com/help/decoy_help.html) [53]. Since the Potato Genome Project are from the diploid *Solanum phureja* and we used a tetraploid potato, the UniProt *Solanum* sequences were included to increase the number of identifications. Search tolerances were 7 ppm for precursors and 0.5 Da for MS/MS fragments. One missed cleavage was allowed and carbamidomethylation of cysteine residues was used as fixed modification and oxidation of methionines as variable modification. Search results were exported from Mascot as XML, including query level results, with a modification to the export script to include protein accession numbers also for the query (spectrum) level results. All search results, including the top-ranked peptide for each spectrum, were imported to Proteios where q values were calculated using the target-decoy method described by Käll et al. [54]. The search results were then filtered at a peptide-spectrum match *q*-value of 0.01 to obtain a false discovery rate of 1% in the filtered list. For quantitative analysis, a label-free approach based on precursor ion intensities was used [55] with all data processing steps performed within Proteios. MS1 peptide feature detection was performed using Dinosaur [56], while the other data processing steps were performed in Proteios, and subsequent feature matching and alignment between LC-MS/MS runs with a previously described workflow [57]. The resulting peptide data was normalized using Loess-G normalization [58] in the Normalyzer software [59]. The normalized data was analyzed using DanteR [60].

### 3.7. Plant Material

All local, national and international guidelines and legislations have been followed and the required or appropriate permissions and/or licenses for the study has been achieved.

## 4. Conclusions

Comparative quantitative proteomic analysis of PTI and ETI interactions revealed that in the PTI interaction proteins generally related to oxidative and biotic stress were upregulated, while proteins related to photosynthesis were downregulated. Furthermore, proteins related to antimicrobial defense and cell wall degradation were also upregulated. Analysis of the ETI interaction showed upregulation of several proteins that were also identified in the PTI interaction. However, we identified distinct upregulation in proteins related to oxidative stress tolerance and GTP binding proteins associated with heterotrimeric G-protein signaling only in the ETI interactions. In addition, proteins related to phospholipase and oxidative stress tolerance were significantly upregulated in only the ETI interactions, such as a chloroplastic lipocalin and a HSP-70 isoform. This study provides a basis for new mechanistic studies and breeding of sustainable resistance in potato. 

## Figures and Tables

**Table 1:** Upregulated proteins in the membrane-enriched fraction from PTI and their regulation in two ETI conditions at 18 hpi (hours post infiltration). The table shows proteins that are upregulated compared to plants infiltrated with only medium as the control condition. Proteins mentioned here are significantly induced (*p*-value < 0.01).

Protein ID	Protein Name	Regulation in PTI (log2)	Regulation in ETI-IpiO (log2)	Regulation in ETI-AVR2 (log2)
Q9LV84	ABC transporter-like	1.36	0.56	0.79
F4KCG9	Alternative NAD(P)H-ubiquinone oxidoreductase C1	1.34	1.32	1.06
C0Z355	AT1G56070 protein	2.76	2.43	2.88
PGSC0003DMP400043466	ATP synthase 24 kDa subunit, mitochondrial	1.19	0.97	0.87
P29790	ATP synthase gamma chain, chloroplastic (F-ATPase)	4.58	4.2	4.43
PGSC0003DMP400035579	ATP synthase subunit b′, chloroplastic	2.34	2.38	2.82
PGSC0003DMP400010643	ATP synthase subunit beta, mitochondrial	1.03	0.48	0.65
Q42267	Carrier protein	0.96	0.35	0.41
PGSC0003DMP400005278	Chaperonin 21	1.62	1.34	1.63
PGSC0003DMP400000640	Charged multivesicular body protein 2a	4.53	5.03	5.57
P07370	Chlorophyll a-b binding protein 1B, chloroplastic (LHCII type I CAB-1B)	5.43	4.23	3.76
PGSC0003DMP400011729	Conserved gene of unknown function	2.07	1.49	1.8
PGSC0003DMP400020125	Conserved gene of unknown function	0.81	0.73	0.48
PGSC0003DMP400026692	Conserved gene of unknown function	4.16	4.27	5.15
PGSC0003DMP400046123	Conserved gene of unknown function	1.39	0.98	1.22
E2FAG4	COSII_At5g14320	1.15	1.2	1.73
Q9ZWH9	Elongation factor 1-α	0.78	0.35	0.48
Q43775	Glycolate oxidase (EC 1.1.3.15)	1.01	0.18	0.67
PGSC0003DMP400009092	Glyoxisomal malate dehydrogenase	1.65	1.01	1.34
Q9LLE0	Hexose transporter	0.7	0.15	0.24
PGSC0003DMP400035078	Hydrolase, acting on ester bonds	1.91	2.36	2.58
B2D2G3	Hydroxypyruvate reductase (EC 1.1.1.81)	1.25	0.55	0.88
B9JNE9	Insertion sequence transposase protein	2.02	2.43	2.78
Q9ZU46	Leucine-rich repeat receptor-like protein kinase	0.67	0.71	1.12
B3H4K6	Magnesium protoporphyrin IX methyltransferase, chloroplastic	1.39	1.3	1.48
A8MQK3	Malate dehydrogenase (EC 1.1.1.37)	1.5	0.66	0.91
PGSC0003DMP400004574	MAR-binding filament 1	0.67	0.41	−0.14
PGSC0003DMP400020545	NAD-malate dehydrogenase	1.26	0.7	0.92
PGSC0003DMP400002176	Nucleolin	1.06	0.33	1.15
PGSC0003DMP400030492	Oligopeptidase	0.84	0.76	1.14
Q9LYJ5	Pectin lyase-like superfamily protein (Polygalacturonase-like protein)	0.58	0.37	0.62
PGSC0003DMP400001052	Peptidyl-prolyl *cis*–*trans* isomerase	0.82	0.73	0.47
PGSC0003DMP400026173	Peroxidase	5.45	5.21	6.28
PGSC0003DMP400013804	Photosystem II D2 protein	2.67	2.82	2.8
PGSC0003DMP400002084	Protein translocase subunit secA	0.9	0.45	0.84
Q30GS3	Putative ferredoxin NADP reductase	2.05	1.72	2.18
Q38M64	Putative uncharacterized protein	1.11	1.18	1.39
Q0WPJ1	Putative uncharacterized protein similar to At1g65260	0.73	0.5	0.61
Q7FIJ2	Putative uncharacterized protein similar to AT4g09410	1.23	1.28	1.16
Q9LMI1	Ribosomal protein L1p/L10e family (T2D23.8 protein)	5.74	5.77	5.61
PGSC0003DMP400060292	Saccharopine dehydrogenase family protein	1.35	1.57	1.31
PGSC0003DMP400000754	Signal peptidase I	0.63	0.43	0.29
PGSC0003DMP400029941	Succinic semialdehyde dehydrogenase	1.61	0.46	0.88
A7LKN1	TAO1	1.28	1.16	1.26
PGSC0003DMP400012430	Transketolase 1	1.25	1.33	1.18
PGSC0003DMP400042799	Translationally-controlled tumor protein homolog	1.71	1.95	2.36
Q8LG76	Zinc finger protein CONSTANS-LIKE 6	1.52	0.83	1.03

**Table 2:** Upregulated proteins only in the two ETI conditions (ETI-IpiO and ETI-Avr2), 18 hpi in the fraction of the membrane-enriched fraction. The tables show proteins that are upregulated as compared to plants infiltrated with only medium as the control condition. Proteins mentioned here are significantly induced (*p*-value < 0.01).

Protein ID	Protein Name	Degree of Regulation in ETI-IpiO (log2)	Degree of Regulation in ETI-AVR2 (log2)
PGSC0003DMP400026606	2-deoxyglucose-6-phosphate phosphatase	1.07	1.57
PGSC0003DMP400026060	3-β hydroxysteroid dehydrogenase/isomerase family protein	0.83	0.91
PGSC0003DMP400002234	30S ribosomal protein S1, chloroplastic	0.45	0.28
PGSC0003DMP400021930	30S ribosomal protein S20	0.6	0.5
Q2MI62	30S ribosomal protein S3, chloroplastic	0.85	0.55
PGSC0003DMP400051744	30S ribosomal protein S5	1.24	1.33
P93014	30S ribosomal protein S5, chloroplastic	0.75	0.75
Q2MI54	30S ribosomal protein S7, chloroplastic	0.7	0.96
Q84P24	4-coumarate—CoA ligase-like 6	0.95	1.07
PGSC0003DMP400008292	50S ribosomal protein L18, chloroplast	0.84	0.79
PGSC0003DMP400046774	50S ribosomal protein L29, chloroplastic	0.93	1.03
A8MQR4	60S acidic ribosomal protein P0	0.72	1.07
PGSC0003DMP400025031	Amino acid binding protein	0.6	0.53
B9DI38	AT1G05190 protein	0.81	0.7
Q1H555	At3g11510	1.02	1.37
Q2MIJ9	ATP synthase subunit a, chloroplastic (F-ATPase subunit IV)	0.77	1.08
Q2MIB4	ATP synthase subunit b, chloroplastic (ATPase subunit I)	0.77	1.19
Q9XF89	Chlorophyll a-b binding protein CP26, chloroplastic (LHCB5) (LHCIIc)	0.71	0.6
PGSC0003DMP400002042	Chloroplast lipocalin	1.08	0.92
A7XZB8	Chloroplast-localized protein	0.67	0.76
PGSC0003DMP400067062	Conserved gene of unknown function	0.59	0.37
PGSC0003DMP400008394	Conserved gene of unknown function	0.53	0.7
Q2MI70	Cytochrome b6-f complex subunit 4 (17 kDa polypeptide)	0.29	0.53
Q1H537	Divinyl chlorophyllide a 8-vinyl-reductase, chloroplastic (EC 1.3.1.75)	0.55	0.57
Q3HVL1	Elongation factor-like protein	0.44	0.84
PGSC0003DMP400027216	Ethylene-responsive small GTP-binding protein	0.36	0.58
PGSC0003DMP400045639	FKBP-type peptidyl-prolyl cis-trans isomerase 3, chloroplastic	0.75	1.02
P400068995	Glucose-1-phosphate adenylyltransferase	0.83	0.7
PGSC0003DMP400051213	Glyceraldehyde-3-phosphate dehydrogenase B subunit	1.27	1.66
PGSC0003DMP400048842	GrpE protein homolog	1.01	0.72
Q8VZ74	GTP-binding protein Era (GTP-binding protein-like)	1.88	1.73
PGSC0003DMP400000783	Heat shock protein 70-3	0.83	1.19
PGSC0003DMP400030419	Heterogeneous nuclear ribonucleoprotein A1	0.84	1.06
PGSC0003DMP400026401	Immunophilin	1.4	1.56
PGSC0003DMP400046332	Isoform 2 of PsbP 2, chloroplastic	0.36	0.67
PGSC0003DMP400029178	NADH dehydrogenase	0.56	0.5
PGSC0003DMP400026922	NADPH:protochlorophyllide oxidoreductase	0.59	1.97
Q3LG51	Nitrite reductase	0.97	0.74
PGSC0003DMP400034084	OJ991214_12.13 protein	0.19	0.82
PGSC0003DMP400025362	Oxygen-evolving enhancer protein 3-1, chloroplast	0.93	1.33
PGSC0003DMP400015048	Peptidyl-prolyl *cis*–*trans* isomerase	1.05	0.22
Q9SR70	Peptidyl-prolyl *cis*–*trans* isomerase FKBP16-4, chloroplastic	0.87	0.89
PGSC0003DMP400018067	Phospholipase A1	0.41	1.09
PGSC0003DMP400048121	Photosystem I subunit XI	0.54	1.07
P06183	Photosystem II 10 kDa polypeptide, chloroplastic (Light-inducible tissue-specific ST-LS1 protein)	1.65	1.83
PGSC0003DMP400040949	Plastid-lipid-associated protein 13, chloroplastic	0.71	0.76
C7ENV4	Polyubiquitin	0.57	0.87
Q7XAB8	Protein THYLAKOID FORMATION1, chloroplastic	0.6	0.87
Q8S9G3	Putative 16 kDa membrane protein	0.73	0.95
Q9SN01	Putative uncharacterized protein AT4g33080	0.53	0.72
Q9FR30	Ripening regulated protein DDTFR10	0.96	1.35
PGSC0003DMP400000966	Serine-type peptidase	0.54	0.29
PGSC0003DMP400011690	Serine/threonine protein kinase	1.28	0.98
PGSC0003DMP400012365	Structural constituent of ribosome	0.56	0.58
PGSC0003DMP400047959	Superoxide dismutase	1.13	2.22
PGSC0003DMP400009317	Superoxide dismutase	0.87	0.7
PGSC0003DMP400016292	Tetratricopeptide repeat protein, tpr	0.09	0.59
PGSC0003DMP400032278	Thylakoid lumenal 17.4 kDa protein	0.86	0.78
PGSC0003DMP400014505	Tic62 protein	0.74	0.79

## Supplementary Materials

Supplementary materials can be found at http://www.mdpi.com/1422-0067/19/2/538/s1.

## Abbreviations

PTI	PAMP triggered immunity
ETI	Effector triggered immunity
